# Taxonomic Distribution of Neoplasia Among Non-Domestic Felid Species Under Managed Care

**DOI:** 10.3390/ani10122376

**Published:** 2020-12-11

**Authors:** Anneke Moresco, Karina E. Muñoz, Federico Gutiérrez, Leonardo Arias-Bernal, Enrique Yarto-Jaramillo, Rodrigo H. F. Teixeira, Juliana Peña-Stadlin, Brigid V. Troan

**Affiliations:** 1 Animal Welfare and Research, Denver Zoo, Denver, CO 80205, USA; 2 Exotic Species Cancer Research Alliance, North Carolina State University, Raleigh, NC 27607, USA; bvtroan@nczoo.org; 3 Patronato del Parque de Las Leyendas, 15088 Lima, Peru; karinafauna@yahoo.com (K.E.M.); fgutierrez57@hotmail.com (F.G.); 4 Bioparque Wakatá, Tocancipá, 251017 Cundinamarca, Colombia; lariasbernal@gmail.com; 5 Programa de Biología, Facultad de Ciencias Universidad El Bosque, 110121 Bogotá, Colombia; 6 Facultad de Medicina Veterinaria, Fundación Universitaria Agraria de Colombia, 111166 Bogotá, Colombia; 7 Zoo de Morelia, Morelia, C.P. 58070 Michoacán, Mexico; eyarto33@hotmail.com; 8 Centro Veterinario México, Ciudad de los Deportes, 03710 Ciudad de México, Mexico; 9 Sorocaba Zoo, R. Vila Hortência, CEP, 18020-268 Sorocaba, Brazil; rhftzoo@hotmail.com; 10 Department of Animal Clinic, Sorocaba University (UNISO) Rod. Raposo Tavares, km 92,5-Vila Artura, 18023-000 Sorocaba, Brazil; 11 Calle 14 Esquina, Zoológico de Cali, 760045 Cali, Colombia; juliana.pena@fzc.com.co; 12 North Carolina Zoo, Asheboro, NC 27205, USA

**Keywords:** cancer, jaguar, mammary carcinoma, Leopardus, Otocolobus, Panthera, Puma, Neofelis, seminoma, Sertoli cell tumor

## Abstract

**Simple Summary:**

Neoplasia in nondomestic felids is common, and frequently malignant. However, few studies include large sample sizes of non-*Panthera* felids. Jaguars are reported to potentially have genetic mutations that predispose them to cancer, but studied populations are limited to North American zoos. By including animals from USA, Mexico, Colombia, Peru, and Brazil, the present study was able to include animals with widely varying genetic backgrounds, diets, and management. This study found that jaguars, lions, tigers and leopards are at a much higher risk of developing cancer than small cats. It also documented that the most common site for neoplasm development is the reproductive tract, followed by the respiratory system and then blood and lymphatic systems. These data provide support for thorough investigation of suspicious lesions to enable early detection of cancer.

**Abstract:**

As evidenced by numerous case reports from zoos, neoplasia in felids is common, but most reports are limited to *Panthera* species in North America or Europe. In order to obtain a wider epidemiologic understanding of neoplasia distribution, necropsy records at seven facilities (USA, Mexico, Colombia, Peru, and Brazil) were evaluated. In contrast to others, this study population (195 cases, 16 species), included many non-*Panthera* felids. Overall neoplasia prevalence was 28.2% (55/195). *Panthera* species had a higher prevalence of neoplasia than non-*Panthera* species (52.5%; vs. 13.0%). Lions (66.7%), jaguars (55.0%), and tigers (31.3%) had the highest species-specific prevalence of neoplasia. Neoplasms in *Panthera* species were more frequently malignant than in non-*Panthera* (86.1% vs. 55.6%). The systems most commonly affected were the reproductive, hematolymphoid, and respiratory. The range of management conditions and more varied genetic backgrounds support a robust taxonomic pattern and suggest that the reported propensity for neoplasia in jaguars may have a genetic basis at a taxonomic level higher than species, as lions and tigers also have high prevalence. Given the high prevalence of neoplasia and high likelihood of malignancy, routine medical exams in all nondomestic felids, but *Panthera* species in particular, should include thorough assessments of any clinical signs of neoplasia.

## 1. Introduction

As medical care improves at zoological institutions, animals live longer and are more likely to develop neoplasia. Comparative studies are a useful tool to learn more about species-specific risk of neoplasia, thereby allowing preventive care, monitoring and treatment to be better tailored for at-risk patients. Better knowledge of risk factors for neoplasia can enhance early detection by simply knowing in which patients and body systems to focus more during each examination. Environmental and genetic factors are known to have an impact on the potential to develop neoplasia; domestic animal studies have shown that certain breeds are more prone to developing neoplasia [1], underscoring the importance of genetic factors. These findings parallel the occurrence of familial neoplasms in humans, such as breast cancer [2]. Similarly, there are specific neoplasms that tend to occur in specific nondomestic felid species, henceforth felids, and reviewed in Terio et al., [3]. In the North American jaguar population, multiple variations in the *BRCA1* sequence were detected, these were traced to common recent ancestors [4].

With some exceptions [5,6,7,8,9,10,11,12], reports of neoplasia in nondomestic felids reflect single cases or case series reports, and the total population of individuals is rarely recorded, making it difficult to estimate prevalence. Additionally, much of the searchable and available literature on nondomestic felid neoplasia is limited to animals cared for at one facility or to the populations in the United States, Canada or Europe [5,9,12,13,14]. Most of these studies focus on *Panthera* felids, while large datasets for non-*Panthera* felids are not reflected in the literature. Even some of the most robust comparative oncology studies include few non-*Panthera* species and none or small neotropical felid species representation [15,16]. A retrospective study including cases from more geographically diverse regions could provide a more robust comparative dataset, as populations with a wider geographic origin will differ in their genetic backgrounds, environmental conditions, and management.

This retrospective study was performed to characterize the prevalence and types of neoplasia in felids under managed care in institutions across the Americas (USA, Mexico, Colombia, Peru, and Brazil), offsetting the effect of limited genetic lines and institutional management practices. The focus of the present study was on the occurrence and species distribution of neoplasms (epidemiology), rather than the clinical course of the cases. Because much of the literature on felid neoplasia has focused on *Panthera* felids, this study also analyzed the prevalence difference between *Panthera* and non-*Panthera* felids. Finally, the prevalence of reproductive cancers is specifically highlighted because diseases of the reproductive organs can have a negative impact on conservation by affecting reproduction. This can be true even if the animal survives when neoplasia is successfully treated by surgical removal of the reproductive organs.

## 2. Materials and Methods

Medical records that included necropsy results for felids were obtained from seven institutions across North and South America (Table 1 and Table 2). Only cases with histologically confirmed neoplasia diagnoses on antemortem or postmortem exam were included. Age was calculated using the date of birth and the date of diagnosis. For animals where the diagnosis was made during postmortem examination (and had not been detected antemortem), age was calculated using date of birth and date of death. For animals that entered managed care via confiscation or rescue, ages were calculated based on estimated ages at the time of accession. Presence of neoplasia was analyzed as a binomial variable (Y = confirmed or N = not detected). Sex, primary tumor location, and type were documented for each neoplasm. Parity was not analyzed as this information was unavailable for any cases that were admitted as confiscations. Histopathological diagnoses were reviewed by one of the authors (BT). Neoplasia nomenclature and classifications were standardized according to organ and cell type (Supplementary Table S1) using the scheme developed by the Exotic Species Cancer Research Alliance (https://escra.cvm.ncsu.edu/) [17], which was based on the National Cancer Institute Thesaurus (https://ncithesaurus.nci.nih.gov/ncitbrowser/) and established veterinary nomenclature in Tumors of Domestic Animals [18]. As has been previously grouped [9], and recognizing the association of reproductive hormones with the development of mammary tumors, neoplasias of the mammary gland, uterus, gonads, and male accessory sex glands were included in the “Reproductive” category.

Since different species have different median life expectancies (MLE), patient ages were analyzed as absolute age and as age relative to MLE (%MLE). When available, sex specific MLEs were used. Maximum recorded lifespan was not used as it represents outliers rather than what may be typical for that species [19]. Values for MLE for all species except *Puma yagouaroundi*, *Leopardus tigrinus*, *L. wiedii* and *L. colocolo* are based on Che-Castaldo et al. [19], currently the only available source for MLE in nondomestic species. For *L. colocolo*, MLE was calculated following Che-Castaldo et al. [19]: ages at the time of death (taxon report, M Dulaney, pers. comm. [20]) were used to calculate a survival curve (GraphPad, Prism, version 9.0.0 (121), San Diego, CA 92108, USA, https://www.graphpad.com/) with age at which half the population remained (after excluding all animals that did not reach 1 year of age) representing the MLE (4370 days = 12.0 years). Data to calculate such survival curves were not available for *Puma yagouaroundi*, *L. tigrinus* or *L. wiedii*, thus these species were excluded from comparisons involving %MLE. Individuals under 12 months of age were also excluded from analysis, to exclude perinatal deaths.

### Statistical Analysis

Data sets were tested for normality with the D’Agostino−Pearson test. Normal data were analyzed and are presented with parametric statistics; data that did not pass the test for normality were analyzed and presented with nonparametric statistics. The level of significance used in all tests was *p* < 0.05. Prevalence of neoplasia between groups was compared with Fisher’s exact test, and continuous variables were compared with Mann−Whitney test. Where pertinent, odds ratios (OR) and confidence intervals (CI) are reported. Multiple logistic regression was used to determine the best model for predicting the development of neoplasia. Commercial statistical software was used (GraphPad, Prism, version 9.0.0 (121), San Diego, CA 92108, USA, https://www.graphpad.com/).

## 3. Results

### 3.1. Neoplasia Type

The records review yielded 271 felids, but after excluding animals younger than 1 year of age and animals for which not enough information was available, a total of 195 felids (91 males and 104 females) from 16 species (Table 2) were included in the present study. Among these 195 felids, 55 were diagnosed with neoplasia, an overall prevalence of 29.2%. A total of 61 unique neoplasias were identified of which 77.0% (47/61) were malignant; six patients (a jaguar, a lion, a tiger, a puma, and two clouded leopards) each had two distinct neoplasms (Supplementary Table S2).

The reproductive system (reproductive organs + mammary glands) was the most commonly affected system overall, 21.3% (13/61) of neoplasms (11 female, 2 male). Seven of eleven neoplasms in females were mammary and one was ovarian (all eight of which were malignant); the three remaining reproductive neoplasms in females were benign uterine leiomyomas. Both male reproductive neoplasms were malignant (seminoma and malignant Sertoli cell tumor). The number of felids known to have been treated with exogenous hormones was small (n =13) and hormone treatment was varied; statistical analysis for association with neoplasia was not possible. Exogenous hormone exposure included melengestrol acetate (MGA; n = 8), deslorelin and MGA (n = 1), depot medroxyprogesterone acetate (n = 1), progestagen treatment (specifics were not included in the medical record, n = 1), and treated “with hormones” for oocyte collection (n = 1). Among the progestin (MGA, DMPA and “progestin”) treated females (n = 11), two developed mammary adenocarcinoma and one a uterine leiomyoma.

Only three of the eleven females with reproductive neoplasms were known to have been treated with exogenous progestins (two mammary gland adenocarcinomas and one uterine leiomyoma), while four mammary, one ovarian sarcoma, one uterine and one vaginal neoplasia cases had not been treated with exogenous hormones. Neither of the males had been exposed to exogenous hormones.

The second most commonly affected system was the hematolymphoid (such as lymphoma/leukemia; 11/61), all but one of which were malignant, and the third most commonly affected system was the respiratory tract (10/61); seven of these neoplasms were lower respiratory, and all but one were malignant. Only one lion with pulmonary carcinoma presented with concurrent anthracosis.

### 3.2. Age, Sex and Taxonomy

Binomial logistical regression models indicated that sex was not a significant variable. Age was a significant variable (*p* = 0.0001), and models with age in absolute numbers (years) were a better fit than age as %MLE, this is likely due to the number of individuals that are not included in the latter model due to the unavailability of MLE, thus it was not possible to calculate %MLE in those individuals. *Panthera* vs. non-*Panthera* classification was a significant variable (*p* < 0.0001), with *Panthera* felids having an estimated odds ratio of 5.4 over non-*Panthera* felids to develop neoplasia. Specific comparisons are outlined below.

#### 3.2.1. Age

Ages (absolute and as a percentage of MLE) for neoplasia and non-neoplasia groups did not pass the test for normality. Age ranges for the neoplasia and the non-neoplasia groups were fairly wide but median age at the time of death/diagnosis was significantly different (Mann−Whitney, *p* < 0.0001) between the two groups (16.0 years (1.7–24.0) and 10.0 years (1–22.9), respectively). Median age (as %MLE) was also significantly lower (Mann−Whitney, *p* = 0.0053) in the non-neoplasia group than the neoplasia group (74.9% and 100.6% resp). Among neoplasia cases, only seven animals were less than 10 years old: a 1.7-year-old Pallas’ cat with urothelial (transitional cell carcinoma), a 3.4-year-old tiger with leukemia, a 3.7-year-old jaguar with lymphosarcoma, a 5.1-year-old lion with leukemia, a 7-year-old lion with melanoma, a 7.6-year-old puma with hemangiosarcoma, and an 8-year-old puma with meningioma. All of these animals were at or under 50% MLE. Ages as %MLE were available for 80 *Panthera* and 78 non-*Panthera*. There was no significant difference in age (%MLE) between the *Panthera* (91.7%) and non-*Panthera* (88.3%) group (Mann−Whitney test, *p* = 0.6402). Age (%MLE) was not significantly different between *Panthera* and non-*Panthera* patients (Mann−Whitney and *p* = 0.7164).

#### 3.2.2. Sex

There was no sex bias in the prevalence of neoplasia among males and females. The male:female ratio in the general study population was not significantly different from the ratio in the subpopulation with neoplasia (Fisher’s exact test, *p* = 0.6505). Neoplasia in females included a significantly larger (Fisher’s exact test *p* = 0.0266) percentage of reproductive system neoplasias than in males (32.4% (11/34) vs. 7.04% (2/27), OR 5.9878; 95% CI 1.354–28.58).

#### 3.2.3. Taxonomy

A significantly higher proportion of *Panthera* patients were affected by neoplasia than non-*Panthera* patients [50.0% (40/80) vs. 13.0% (15/115)]; Fisher’s exact test *p* < 0.0001) (Table 3), OR 7.407 (95% CI 3.61 to 15.14). Among *Panthera* felids, 45.0% (36/80) developed malignant neoplasia whereas only 8.7% (10/115) among non-*Panthera* felids did, this difference was significant (Fisher’s exact test *p* < 0.0001; OR 8.591, 95%CI 3.94 to 18.07). In contrast, the percentage of *Panthera* and non-*Panthera* patients that developed benign neoplasms was not significantly different (Fisher’s exact test *p* =0.7743): 5.0% (4/80) vs. 4.4% (5/115). When the analysis was performed based on the neoplasms, rather than the patients, the percentage of malignant neoplasms among *Panthera* patients (86.1%; 37/43) was significantly higher (Fisher’s exact test *p* = 0.0425) compared to non-*Panthera* (61.1%; 11/18).

Lions and jaguars were over-represented in the neoplasia group as together they comprised 56.4% (31/55) of neoplasia cases, while only comprising 27.2% (53/195) of the total study population. Among species with 10 or more individuals included in this study population, the three highest species-specific prevalence of neoplasia were: lions (60.1%; 20/33), jaguars (55.0%; 11/20), and tigers (31.3% 5/16). Jaguars had the highest species-specific reproductive neoplasia prevalence (20.0%; 4/20), followed by tigers (18.8%; 3/16), and lions (9.1%; 3/33); the overall study population reproductive neoplasia prevalence was 7.2% (14/195). Prevalence of reproductive neoplasia was lower in non-*Panthera* [3.5% 4/115)] than in *Panthera* [12.5% (10/80)]. This difference was significant (Fisher’s exact test *p* = 0.0228; OR 3.964, 95% CI 1.263 to 11.76).

## 4. Discussion

The present study systematically summarizes a large dataset of neoplasia in *Panthera* and non-*Panthera* felid species. The most striking findings were the significantly higher neoplasia prevalence in the *Panthera* vs. non-*Panthera* felids, the higher probability of malignancy among *Panthera* compared to non-*Panthera* felids, and the higher prevalence of reproductive neoplasia among *Panthera*. The occurrence of neoplasia is associated with increasing age, but age does not explain this difference as it was not significantly different between *Panthera* and non-*Panthera*.

Jaguars in particular have been reported to be prone to reproductive neoplasia, possibly related to *BRCA1* mutations, but these mutations were traced back to common ancestors within the North American jaguar population. Mutations of *BRCA1* genes are associated with increased reproductive tumors in humans [2,21] mutations that may predispose animals to reproductive neoplasms have not been investigated in felids other than jaguars. The felids included here originate from a wide geographic range and therefore represent varied genetic lineages as well as diverse environmental and management conditions. That is, the high neoplasia prevalence among *Panthera* seems to transcend recent genetic lines (ex situ breeding) and management practices.

The present study found a high prevalence of reproductive neoplasia in jaguars, and tigers, which is similar to a study focused on mammary neoplasia, where jaguars and tigers were over- represented [7]. Sample size for leopards and snow leopards in the present dataset was low (less than n = 10). However combining the present data for jaguars, lions, tigers, leopards and snow leopards, with data from Kloft et al. [9], it is possible to obtain a combined dataset where all five species have n > 10, and high neoplasia prevalence is present for all *Panthera* species except snow leopards: jaguars (57.1%; 12/21), leopards (55.6%, 10/18); lions (53.5%, 46/86), tigers (48.7%, 75/154) and snow leopards (16.7%, 2/12). The lower prevalence of neoplasia in non-*Panthera* felids in this dataset is consistent with a previous report focused on mammary neoplasia which reported that non-*Panthera* felid species develop mammary neoplasia less frequently than *Panthera* species [7].

Although a high percentage of female jaguars in the study population developed neoplasia (70.0%, 7/10), none was reported to have malignant ovarian carcinomas, a neoplasm previously reported in multiple jaguars from the North American population [6]. This discrepancy is likely due to the founder effect in North American jaguars as mentioned above. Data from the Reproductive Health Surveillance Program (RHSP) show that jaguars [80.0% (28/35)], leopards [54.2% (13/24)], tigers [41.1% (30/73)], and lions [32.5% (25/77)] all have a high species-specific prevalence of female reproductive system neoplasia (Reproductive Health Surveillance Program, Moresco and Agnew pers. comm. [22]), other North America-based studies have shown similar results [9,13]. In the present study females comprised the majority of reproductive neoplasia (85.7%, 12/14) and mammary cancer was the most common reproductive neoplasia. It is known that in felids reproductive neoplasia, mammary in particular, tends to be malignant [6,7,23,24]. Concordantly, all cases of mammary, and most cases of reproductive neoplasia in this study were malignant; additionally, the majority of all neoplasms of the reproductive system were also malignant. A malignant Sertoli cell tumor was reported in this study, no reproductive neoplasia had been found in male jaguars in past surveys [5,25]. Reproductive disease can impact reproduction and thereby conservation, the age at which reproductive neoplasia was diagnosed tended to be in animals that would be able to breed if given the opportunity, the youngest animal with reproductive neoplasia was 11 years old.

Hematolymphoid neoplasia prevalence in this study population was high; however this is not remarkable as these are very common across veterinary species. Prevalence studies in cats and dogs indicate that lymphoma is one of the most common neoplasias [26,27]. Hematolymphoid neoplasias also appear common in nondomestic felids [9,28]; however, in contrast to domestic cats, many cases in nondomestic felids are not associated with retroviruses.

The high prevalence of respiratory tract neoplasia is notable as lung neoplasia in nondomestic felid species is rarely documented in general surveys [11] (B. Troan pers. comm. [29]) and remains relatively uncommon [9,18,30]. Respiratory neoplasias were diagnosed in four species in this study. Seven of ten neoplasms were in *Panthera* (four lions, three jaguars) and three were non-*Panthera* (two Pallas’ cats and one clouded leopard), with cases reported from four different facilities. A decrease in lung cancer risk with increasing altitude has been reported for humans [18]; however half of the cases were reported by facilities at low altitudes (400 meters and 96 meters). Anthracosis has been reported to be associated with malignant lung neoplasia in domestic dogs [31], assessing such an association was not possible in this dataset as there were only seven cases of lower respiratory neoplasia and only one of those presented with anthracosis.

## 5. Conclusions

In conclusion, the data presented here demonstrate that felids in the *Panthera* genus have a higher risk of developing neoplasia than non-*Panthera* felids. Although non-*Panthera* felids have a lower relative risk of developing neoplasia, all felids seem to have a propensity to develop malignant neoplasia. High neoplasia prevalence was observed in *Panthera* populations over wide ranges of latitude and altitude, management, dietary sources, and familial lines. These data support the possibility of a genetic predisposition or propensity at the genus or higher level, and may be compounded by mutations from a few over-represented individuals in zoo populations. Such trends could be evaluated within the context of the evolution of felids [32]. Although this study did find high prevalence of malignant neoplasia in jaguars, malignant ovarian neoplasia was not a salient feature. This is in contrast to findings in the North American zoo population [6,33], supporting the idea that the predisposition to malignant ovarian tumors may be associated with additional mutations introduced to a closed ex situ population.

The amount of data this study gathered underscores the overall value of large collaborative international studies as well as the value of well-established necropsy protocols and searchable record systems. Based on these findings, felids (in particular *Panthera*), should be regularly monitored for neoplasia development, as early detection will be key to successful management of neoplasia that will most likely be malignant. In addition to regular, thorough physical exams, new noninvasive technology, such as thermography can help detect and monitor superficial neoplasia in nondomestic species [34]. Finally, further research in non-*Panthera* felids under managed care in range countries is warranted to improve knowledge about the specific causes of death, as these species remain underrepresented in the literature.

## Figures and Tables

**Table 1:** Institutions and cases contributed to the survey of felid neoplasia.

Institution	Location	Cases
Denver Zoo	Denver, USA	50
North Carolina State University	Raleigh, USA	13
Parque de las Leyendas	Lima, Perú	36
Bioparque Wakatá	Tocancipá, Colombia	13
Morelia Zoo	Morelia, México	5
Zoologico de Cali	Cali, Colombia	59
Sorocaba Zoo	Sorocaba, Brazil	19
Total		195

**Table 2:** List of species included in the survey of neoplasia in felids from zoos in the US, Mexico, Peru, Colombia, and Brazil.

Common Name	Scientific Name	Female (n)	Male (n)	MLE ^†^ (Years)
**non-*Panthera***				
Cheetah	*Acinonyx jubatus*	1	1	11.6 ^a^
Pampas cat	*Leopardus colocolo*	1	1	12.0 ^b^
Ocelot	*Leopardus pardalis*	13	7	15.4 ^a^
Tigrina	*Leopardus tigrinus*	5	7	NA
Margay	*Leopardus wiedii*	7	5	NA
Serval	*Leptailurus serval*	2	2	13.2 ^a^
Canadian lynx	*Lynx canadensis*	0	1	15.0 ^a^
Clouded leopard	*Neofelis nebulosa*	3	2	13.1
Pallas’ cat	*Otocolobus manul*	6	13	8.5 ^a^
Puma	*Puma concolor*	15	13	M 13; F 16 ^a^
Jaguarundi	*Puma yagouaroundi*	4	6	NA
non-*Panthera* total		57	58	
***Panthera***				
Lion	*Panthera leo*	19	14	16.9 ^a^
Jaguar	*Panthera onca*	10	10	17.8 ^a^
Leopard	*Panthera pardus*	3	1	18.2 ^a^
Tiger	*Panthera tigris*	11	5	M 16; F 14.3 ^a^
Snow leopard	*Panthera uncia*	4	3	15.1 ^a^
*Panthera* total		47	33	
Grand total		104	92	196

^†^ MLE = Median life expectancy, NA = not available; M= male, F = female; ^a^ Che-Castaldo et al. [19]; ^b^ calculated from Pampas cat Taxon Report (M. Dulaney pers. comm. [20])

**Table 3:** Prevalence of neoplasia by species in felids in managed care.

Species	No Neoplasia	Neoplasia Cases	Total	Prevalence (%)
**Non-*Panthera***				
*Acinonyx jubatus*	2	0	2	0
*Leopardus colocolo*	2	0	2	0
*Leopardus pardalis*	17	3	20	15.0
*Leopardus tigrinus*	11	1	12	8.3
*Leopardus wiedii*	12	0	12	0
*Leptailurus serval*	4	0	4	0
*Lynx canadensis*	1	0	1	0
*Neofelis nebulosa*	2	3	5	60.0
*Otocolobus manul*	16	3	19	15.8
*Puma concolor*	24	4	28	14.3
*Puma yagouaroundi*	9	1	10	10.0
Total Non-*Panthera*	100	15	115	13.0
***Panthera***				
*Panthera leo*	13	20	33	60.1
*Panthera onca*	9	11	20	55.0
*Panthera pardus*	2	2	4	50.0
*Panthera tigris*	11	5	16	31.3
*Panthera uncia*	5	2	7	28.6
Total *Panthera*	40	40	80	50.0
Grand Total	140	55	195	28.2

## Supplementary Materials

The following are available online at https://www.mdpi.com/2076-2615/10/12/2376/s1. Table S1: Histopathology classification scheme; Table S2: Primary neoplasia cases in felids across the Americas.
