# Supplementary material for: Taxonomic Distribution of Neoplasia Among Non-Domestic Felid Species Under Managed Care
Source: Animals (Basel). 2020 Dec 11;10(12):2376. doi: 10.3390/ani10122376 (PMC7763325; doi:10.3390/ani10122376)
Supplement: Supplementary file 1 [file animals-10-02376-s001.pdf]

Supplemental Table 1. Histopathology classification scheme. This table corresponds with Moresco et al., Taxonomic distribution of neoplasia among nondomestic felid species under managed care. *Animals*.

System	Organs/Cell Types Included
Alimentary	oral cavity<br>gastrointestinal tract<br>exocrine pancreas<br>hepatobiliary system including gall bladder
Endocrine	adrenal gland<br>thyroid<br>parathyroid<br>endocrine pancreas
Hematolymphoid	bone marrow<br>immune cells origin (e.g., lymphocyte, mast cell, histiocyte)<br>spleen
Mesothelium	tumors of mesothelium (pleura, peritoneal)
Nervous	brain<br>spinal cord<br>peripheral nervous system<br>meninges
Reproductive	ovary/testes<br>uterus (including fallopian tubes)<br>mammary gland<br>male accessory sex organs
Respiratory	upper respiratory tract (sinus and nasal passages)<br>trachea<br>lungs
Skin	integument (including eyelid and external ear)<br>cutaneous pigment cell tumors
Soft tissue	soft tissue/spindle cell tumor of connective tissue (not specified)<br>muscle<br>adipose<br>fibrous tissue<br>vascular
Urinary	kidney<br>ureter<br>bladder

Neoplasia nomenclature and classifications were standardized according to organ and cell type using the scheme developed by the Exotic Species Cancer Research Alliance (https://escra.cvm.ncsu.edu/), which was based on the National Cancer Institute Thesaurus (https://ncithesaurus.nci.nih.gov/ncitbrowser/) and established veterinary nomenclature in Tumors of Domestic Animals [Meuten].

Supplemental Table 2. Primary neoplasia cases in felids across the Americas. This table corresponds with Moresco et al., taxonomic distribution of neoplasia among nondomestic felid species under managed care. *Animals*.

Species	Case Number	Sex	age (yr)	System	Diagnosis	Type†
**Non-Panthera**						
Leopardus pardalis	35	M	17.9	Alimentary	pancreatic adenocarcinoma	M
Leopardus pardalis	7	F	21.7	Reproductive	uterine leiomyoma	B
Leopardus pardalis	32	F	24.0	Skin	squamous cell carcinoma	M
Leopardus tigrinus	27	F	17.0	Reproductive	ovarian sarcoma	M
*Neofelis nebulosa**	22	M	15.0	Endocrine	pheochromocytoma	B
Neofelis nebulosa	51	F	18.7	Endocrine	thyroid adenoma	B
*Neofelis nebulosi**	37	F	14.4	Hematolymphoid	mast cell tumors	B
*Neofelis nebulosa**	37	F	14.4	Urinary	urothelial carcinoma	M
*Neofelis nebulosa**	22	M	15.0	Respiratory	bronchogenic papillary carcinoma	M
Otocolobus manul	6	M	10.9	Respiratory	nasal adenocarcinoma	M
Otocolobus manul	9	M	10.2	Respiratory	bronchoalveolar adenoma	B
Otocolobus manul	4	M	1.7	Urinary	urothelial carcinoma of the ureter	M
Puma concolor	50	M	14.1	Alimentary	oral squamous cell carcinoma	M
Puma concolor	16	F	8.0	Nervous	meningioma	B
*Puma concolor**	20	M	19.0	Reproductive	seminoma	M
*Puma concolor**	20	M	19.0	Skin	basal cell carcinoma	M
Puma concolor	33	F	7.0	Vascular	hemangiosarcoma	M
P. yagouaroundi	28	M	13.3	Soft tissue	soft tissue sarcoma	M
**Panthera**						
Panthera leo	21	F	10.0	Alimentary	bile duct carcinoma	M
Panthera leo	45	M	17.4	Alimentary	hepatic carcinoma	M
Panthera leo	52	M	14.0	Alimentary	hepatocellular carcinoma	M
*Panthera leo**	25	M	17.0	Endocrine	adrenal cortical adenoma	M
Panthera leo	42	F	19.0	Endocrine	adrenal cortical carcinoma	M
Panthera leo	3	F	18.2	Hematolymphoid	lymphosarcoma	M
Panthera leo	5	F	11.8	Hematolymphoid	splenic lymphosarcoma	M
Panthera leo	13	F	12.0	Hematolymphoid	histiocytic sarcoma	M
Panthera leo	31	M	16.0	Hematolymphoid	lymphoma	M
Panthera leo	34	M	5.1	Hematolymphoid	leukemia	M
Panthera leo	46	M	15.2	Hematolymphoid	anaplastic T cell lymphoma	M
Panthera leo	2	F	18.3	Reproductive	uterine leiomyoma	M
Panthera leo	12	F	15.5	Reproductive	mammary adenocarcinoma	M
Panthera leo	40	F	16.0	Reproductive	mammary adenocarcinoma	M
Panthera leo	11	F	11.0	Respiratory	lung adenocarcinoma	M
Panthera leo	15	F	12.0	Respiratory	pulmonary sarcoma	M
Panthera leo	38	F	17.2	Respiratory	pulmonary carcinoma	M
Panthera leo	47	F	21.0	Respiratory	pulmonary carcinoma	M
Panthera leo	54	M	18.6	Respiratory	bronchoalveolar carcinoma	M
Panthera leo	1	M	7.0	Skin	eyelid melanoma	M
*Panthera leo**	25	M	17.0	Urinary	bladder urothelial carcinoma	M
Panthera onca	18	F	18.0	Alimentary	pancreatic adenocarcinoma	M
Panthera onca	8	F	19.7	Hematolymphoid	lymphoma	M
*Panthera onca**	44	M	16.0	Hematolymphoid	lymphoma	M
Panthera onca	53	F	3.7	Hematolymphoid	lymphosarcoma	M
Panthera onca	10	F	18.1	Reproductive	mammary adenocarcinoma	M
Panthera onca	43	F	20.6	Reproductive	solid mammary carcinoma	M

Panthera onca	49	F	17.5	Reproductive	mammary carcinoma	M
Panthera onca	55	M	20.2	Reproductive	malignant Sertoli cell tumor	M
Panthera onca	17	M	18.3	Respiratory	nasal squamous cell carcinoma	M
Panthera onca	26	F	16.4	Respiratory	bronchial adenocarcinoma	M
Panthera onca	41	M	20.0	Soft tissue	soft tissue sarcoma	M
*Panthera onca**	44	M	16.0	Vascular	splenic hemangioma	B
Panthera pardus	29	M	16.7	Endocrine	thyroid adenoma	B
Panthera pardus	23	F	18.1	Soft tissue	lipoma	B
*Panthera tigris**	19	F	11.0	Endocrine	thyroid carcinoma	M
Panthera tigris	36	M	3.4	Hematolymphoid	leukemia	M
Panthera tigris	14	F	15.0	Mesothelial	malignant mesothelioma	M
*Panthera tigris**	19	F	11.0	Reproductive	uterine leiomyoma	B
Panthera tigris	24	F	15.1	Reproductive	mammary adenocarcinoma	M
Panthera tigris	30	F	19.5	Reproductive	mammary adenocarcinoma	M
Panthera uncia	48	F	13.8	Alimentary	oral squamous cell carcinoma	M
Panthera uncia	39	M	15.1	Endocrine	thyroid adenoma	B

* animals with two distinct neoplasias are listed twice, once for each neoplasm † M = malignant; B = benign
